# When language engenders discomfort

**DOI:** 10.1111/medu.15598

**Published:** 2025-01-04

**Authors:** Justin P. Boyle, Justin L. Bullock

**Affiliations:** ^1^ General Internal Medicine University of Toronto Toronto Ontario Canada; ^2^ School of Health Professions Education Maastricht University Maastricht the Netherlands; ^3^ Nephrology University of Washington School of Medicine Seattle Washington USA

## Abstract

Language holds power & can harm: @justin_p_boyle and Justin Bullock explore the cisheteropatriarchy and epistemic violence regarding language in #MedEd.

We must be mindful of the language we use when caring for patients. This is regularly emphasized in medical education curricula, particularly when teaching trainees how to practice cultural humility and trauma‐informed care for patients from marginalized communities. It is, therefore, simultaneously troubling and unsurprising to read ‘Beyond Inclusion Politics: A Critical Discourse Analysis of Sex and Gender in Medical Education’.^1^ In it, Kariyawasam et al. report, through an autoethnographic examination of a preclinical undergraduate medical school curriculum, that the everyday use of ill‐defined gendered and sexed language was not only present but also found to uphold systems of transphobia and cisheteropatriarchy within medicine. Here, we explore our own reactions to the authors' use of language because these reactions emphasize important aspects of their conclusions.

In the spirit of autoethnographic introspection, we felt a sense of discomfort when first reading this autoethnography because of the way in which the authors powerfully wielded language. Their words pierced our fragile academic skin: ‘Trans and intersex bodies cannot be considered addendums to be tacked on to a foundation of cisnormative and inaccurate teaching’.^1^ Reading the article, we found ourselves attempting to appease our discomfort by interrogating the piece's methodologic rigour, looking for tables that clearly documented exactly which words the autoethnographer had recorded. This level of seeking word‐for‐word proof was unfair: it is not a practice which we typically undertake or expect of other manuscripts. Unsettled by our own discomfort, we asked ourselves, ‘Where is this disconcertion coming from?’ As we share our journey to answer this question, we would be remiss not to acknowledge that we, two queer, cisgendered individuals are permitted to comment on a piece describing the repetitive harms of sexed and gendered language on trans and gender diverse individuals in medical education. Upon reflection, we realized that the discomfort we felt during our initial review of this autoethnography is likely rooted in the politics of our identities and the power that comes from the language we use. Written plainly, the authors did not write like we do. We never questioned the authors' core findings, but rather, we questioned whether this piece was appropriate for this academic setting.

In the spirit of autoethnographic introspection, we felt a sense of discomfort when first reading this autoethnography

Because the authors so directly critiqued the cisheteropatriarchy in medical education, we defensively hid behind assessing methodology as a gatekeeping tool to determine whether this article was worthy of joining our academic dialogue. We were empowered as if we were the gatekeepers of this academic dialogue. In so doing, we committed an act of epistemic violence through our reflexive actions to judge the legitimacy of this work. Epistemic violence refers to the active oppression and displacement of non‐dominant communities from knowledge‐creating structures to suppress their political voices.^2^ Such active oppression is exclusionary and undermines a non‐dominant group's ability to be heard. By oppressing diversity of voice, epistemic violence controls language and how the realities of marginalized communities are communicated and valued. It is ironic, therefore, that we found ourselves reflexively engaged in epistemic violence in reaction to Kariyawasam et al.'s demonstration that language poses substantial harms by perpetuating misogyny and normative gender identities in medical education. The article, as a result, provides a very useful reminder that the language we use is the foundation of the knowledge we learn. In the case of medical education, it is reflective of those dominant groups that historically and presently hold power (i.e. the cisheteropatriarchy). Overcoming our initial misgivings, therefore, requires further reflection on a variety of critical questions: What is the cisheteropatriarchy to which Kariyawasam et al. attribute substantial harm? Was the discomfort that we were feeling a manifestation of the cisheteropatriarchy of medical education? What is the relationship between epistemic violence and the cisheteropatriarchy?

epistemic violence controls language and how the realities of marginalized communities are communicated and valued

The cisheteropatriarchy, built upon conceptualizations from Black feminist scholars such as bell hooks, who critiqued the Imperial White Supremacist Capitalist Patriarchy, is a system of power that places cis‐gender, straight white males as both superior and normative in their expression of gender and sexuality.^3^ This system is rooted in the assumption that the superior way of being is that of the dominant racial, sexual and gender group. This leads to the continued sociocultural and institutional marginalization of non‐dominant communities in medical education and medicine broadly, especially trans and gender diverse individuals.^4^, ^5^, ^6^ In the context of medical education, this institutionalized oppression is enforced by epistemic violence.

Critiquing the cisheteropatriarchy requires every person to examine those aspects of the cisheteropatriarchy within themselves. We, as two queer men, do not identify as belonging to the cishetereopatriarchy, and yet, our reaction to Kariyawasam et al.'s article shows that we clearly still uphold cisheteropatriarchical norms. We agree with the authors that the very educational framework in which physicians are trained commits epistemic violence at the expense of those from non‐dominant communities. Language is fundamental to this epistemic enforcement and the policing of language maintains the cisheteropatriarchy.

Critiquing the cisheteropatriarchy requires every person to examine those aspects of the cisheteropatriarchy within themselves.

This autoethnography has inspired us to critically reflect on the ways that medical educators weaponize language in harmful ways. We have seen within ourselves how our everyday utterances and academic gatekeeping actively harm others in our community. Language can covertly uphold the cisheteropatriarchy and epistemic violence in medical education. As much as this study is a call to examine language in external structures that perpetuate systems of transphobia and cisheteropatriarchy, it is also important to be aware of how language can act as a mirror, outwardly reflecting both the conscious and unconscious biases we hold. Critically examining language can help to highlight those aspects of the cisheteropatriarchy that can be hard to see. By engendering discomfort, language empowers us to understand our positionality and confront biases and oppression within medical education.

language can act as a mirror, outwardly reflecting both the conscious and unconscious biases we hold

## AUTHOR CONTRIBUTIONS

**Justin P. Boyle:** Conceptualization; writing—original draft. **Justin L. Bullock:** Conceptualization; writing—original draft.
